# Biological and molecular profile of fracture non-union tissue: current insights

**DOI:** 10.1111/jcmm.12532

**Published:** 2015-03-01

**Authors:** Michalis Panteli, Ippokratis Pountos, Elena Jones, Peter V Giannoudis

**Affiliations:** a Academic Department of Trauma & Orthopaedics, School of Medicine, University of LeedsLeeds, UK; b Leeds Institute of Rheumatic and Musculoskeletal Medicine, School of Medicine, University of LeedsLeeds, UK; c NIHR Leeds Biomedical Research Unit, Chapel Allerton HospitalLS7 4SA Leeds, West Yorkshire, UK

**Keywords:** non-union(s), human tissue, bone morphogenic protein(s), mesenchymal stem cell(s)

## Abstract

Delayed bone healing and non-union occur in approximately 10% of long bone fractures. Despite intense investigations and progress in understanding the processes governing bone healing, the specific pathophysiological characteristics of the local microenvironment leading to non-union remain obscure. The clinical findings and radiographic features remain the two important landmarks of diagnosing non-unions and even when the diagnosis is established there is debate on the ideal timing and mode of intervention. In an attempt to understand better the pathophysiological processes involved in the development of fracture non-union, a number of studies have endeavoured to investigate the biological profile of tissue obtained from the non-union site and analyse any differences or similarities of tissue obtained from different types of non-unions. In the herein study, we present the existing evidence of the biological and molecular profile of fracture non-union tissue.

- Introduction
- Materials and Methods
  Eligibility Criteria
  Information Sources
  Study Selection
  Extraction of Data
  Data Analysis
- Results
  Literature Search
  Studies Characteristics
  Macroscopic Structure of non-union Tissue
  Microscopic Structure of non-union Tissue
  Bacteriology of the non-union
  Evaluation of Tissue Sample
  Cultures Characteristics
- Discussion
- Conclusion

## Introduction

Bone healing is a complex but well-orchestrated physiological process which recapitulates aspects of the embryonic skeletal development in combination with the normal response to acute tissue injury 1,2. It encompasses multiple biological phenomena and is margined by the combination of osteoconduction (scaffold formation), osteoinduction (timed cellular recruitment controlled by multiple signalling molecules) and osteogenesis (new bone formation) 2–5. In contrast to the scar formation, which occurs in the majority of other tissue types in adults, bone has the innate capability to repair and regenerate, regaining its former biomechanical and biochemical properties 6–8.

During the bone healing process, a well-regulated series of overlapping processes take place in the cortical bone, the periosteum, the bone marrow and the undifferentiated fascial tissue surrounding the fracture 10,12,13. According to the histological appearance, two basic types of bone healing have been identified 6,7,11. The primary (direct) healing pattern occurs when anatomical reduction is achieved, along with almost absolute stability 3,15. The disrupted continuity of the bone in this type of healing is re-established with regeneration of the Harvesian system and the lamellar bone, with therefore no need of any remodelling 12,15. On the contrary, the secondary (indirect) healing pattern that occurs in the vast majority of clinical cases depends to the formation of fibrocartilaginous callus 3,6. This process can be broadly divided into five stages: that of inflammation, granulation tissue formation, soft callus formation (hyaline cartilage), hard callus formation (woven bone) and remodelling 6,9,11,14.

In more detail, following an injury the bone architecture is disrupted, as is the surrounding soft tissue continuity. Consequently, the local blood vessels are torn, a haematoma is formed and the coagulation cascade is activated 16. This fracture haematoma contains cells that originate from the peripheral and intramedullary blood, as well as from the bone marrow 15. They include inflammatory immune cells, neutrophils, monocytes and macrophages that are activated by the coagulation process; fibroblasts; and mesenchymal stem cells (MSCs) 6,16. Prostaglandins, cytokines and other proteins are abundant in this environment and contribute to the formation of a complex microenvironment which has different effect on each cell population 6. These mediators are known to increase cellular migration, proliferation, enhance osteogenesis, collagen synthesis and angiogenesis 6.

Subsequently, the necrotic or damaged pieces of bone are removed and the fracture haematoma is gradually replaced by granulation tissue 17. The osteoprogenitor cells then proliferate and differentiate, leading to deposition of collagen and formation of soft callus. An increased vascularity and intense cell proliferation in the cambium layer of the periosteum is evident in this stage 13,17. Bone formation then occurs by endochondral or intramembranous ossification. Initially, immature woven bone characterized by coarse collagen fibres arranged in a haphazard fashion is formed, but is then transformed to mature lamellar bone (remodelling) in a slow process 13,17. During remodelling that could last several months to years after fracture, both osteoblast and osteoclast activity is intense, with bone resorption followed by appositional production of new bone by osteoblasts 17.

*In vitro* investigations to evaluate osteogenic activity include measurements of a number of secreted substances (proteins) including: alkaline phosphatase (ALP), osteonectin, osteopontin, osteocalcin and bone sialoprotein. Alkaline phosphatase is a key protein secreted by osteoblasts in response to osteogenic activity and represents a marker of the earlier stage of osteoblast differentiation 18. Osteonectin, osteopontin and osteocalcin are non-collagenous bone matrix proteins, abundant in bone tissue 19. They are thought to be of great importance in bone development, growth, turnover and fracture repair; along with osterix, as essential factor for osteoblast differentiation and bone formation, they represent markers of the later stage of differentiation 18–20. Bone Sialoprotein, an extracellular matrix protein secreted by osteoblastic cells, has also been reported to modulate osteoblast differentiation and mineralization 21.

As already mentioned, the physiological sequence of fracture healing depends on numerous endogenous and exogenous factors 22,23. If this sensitive balance is altered in any way, complications may arise, such as delayed union or non-union. The criteria for defining a non-union are not yet standardized 24. FDA (Food and Drug Administration) defines a non-union as the incomplete fracture healing within 9 months following injury, along with absence of progressive signs of healing on serial radiographs over the course of three consecutive months 25. In the United States alone, it is estimated that 5–10% of all fractures are complicated by non-union or delayed union 26, posing an enormous economic burden to the healthcare system 27. The tibia and the femur are the most common long bones associated with the development of non-union 28,29.

According to the radiological and histological appearance, non-unions are characterized as: hypertrophic, usually resulting from insufficient fracture stabilization (extensive callus formation) 30; and atrophic, where the fracture stabilization is adequate but there is localized dysfunction in biological activity (little callus formation and presence of a fibrous tissue-filled fracture gap) 30,31. Synovial pseudarthrosis is considered as a different pathological entity, caused by inadequate immobilization with or without the presence of infection 32. Moreover, non-unions can be characterized according to the presence of bacteria at the fracture site, as septic or aseptic non-unions 33.

It is generally accepted that the progression to a non-union in most cases represents a multifactorial process. Various risk factors have been implicated with compromized fracture healing, including: patient dependent factors such as age, gender, medical comorbidities (*i.e*. anaemia, diabetes and hormone disorders), smoking and administration of pharmacological agents (*i.e*. steroids, non-steroidal anti-inflammatories, *etc*.); and patient independent factors such as the ‘personality’ of the fracture, presence of infection and adequacy of surgical technique 22,25,34.

The exact biological process leading to a non-union remains obscure and it is well accepted that any planned interventions to reverse this process should be well-timed and well-aimed to restore both biological and mechanical deficiencies 3,14,31,35. It can be postulated that by gaining a better understanding of the underlying mechanisms leading to a non-union, both clinicians and scientists would be allowed to target specific pathways independently, tailoring treatment to each patient's individual requirements 11. Therefore, the purpose of this review is to investigate the biological profile of tissue obtained from the non-union site and to analyse any differences or similarities of tissue obtained from different types of non-unions. Moreover, it aims to evaluate whether any interventions on the tissue obtained would influence in a positive aspect its biological characteristics and bone repair responses.

## Materials and methods

This review was conducted in accordance to the PRISMA guidelines 36. Data were documented according to a standardized protocol, where objectives and inclusion criteria were specified in detail.

### Eligibility criteria

Studies selected were original articles fulfilling the following inclusion criteria: (*i*) the tissue was obtained from a non-union site and examined or processed for defining its characteristics and properties; (*ii*) only tissue acquired from human subjects was included; (*iii*) articles were published in English language and (*iv*) the full text of each article was available. All studies that did not fulfil all eligibility criteria were excluded from further analysis, whereas no publication date restrictions were imposed.

### Information sources

Studies were identified by searching the following resources/databases: PubMed Medline; Ovid Medline; Embase; Scopus; Google Scholar; and the Cochrane Library, to retrieve all available relevant articles. The terms used for the search included: non-union(s), nonunion(s), human, tissue, bone morphogenic protein(s) (BMP's) and MSCs. The identified articles and their bibliographies including any relevant reviews were manually searched for additional potential eligible studies.

### Study selection

Two of the authors (M.P., I.P.) performed the eligibility assessment, in an independent, unblinded and standardized manner. Most citations were excluded on the basis of information provided by their respective title or abstract. In any other case, the complete manuscript was obtained, scrutinized by the two reviewers and included if fulfilling the eligibility criteria. Any disagreement between reviewers was resolved by consensus.

### Extraction of data

Relevant information on author's name, publication year, patient demographics, site and duration of non-union, type of the non-union, characteristics and evaluation of tissue samples, culture properties, gene expression, protein expression and effect of additional interventions was carefully extracted.

### Data analysis

All outcomes of interest were inserted in an electronic database and outcome of different studies were documented. The characteristics of tissue samples were then compared across different studies and the effect of any intervention was evaluated.

## Results

### Literature search

**Fig 1:**
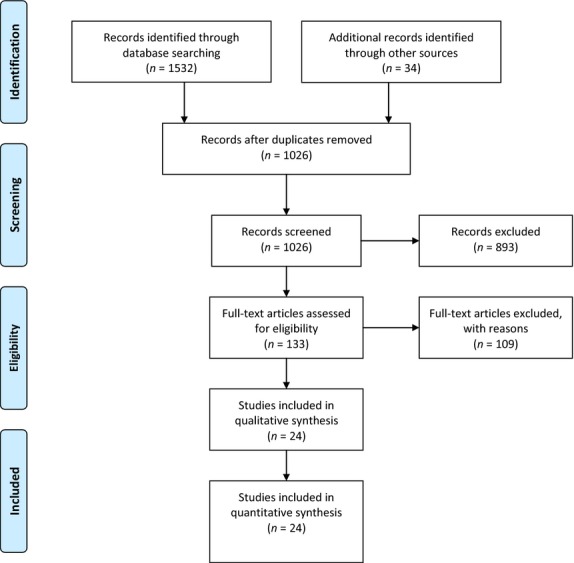
Flow chart of the study selection.

The electronic search of the literature retrieved 1532 citations, but only 21 of them met the selection criteria 14,18,19,22,30,35,37–51. Another three eligible papers 32,52,53 were obtained from the hand search of the references of the eligible studies and relevant review articles, yielding 24 eligible studies for the final analysis (Fig.1) 14,18,19,22,30,32,35,37–53.

All studies were published from 1954 to 2013 and included 467 cases (Table1) 14,18,19,22,30,32,35,37–53. Some of the authors used the same tissue bank for their analysis, but as different investigations were performed in each study, they were included as different studies 14,19,35,39,47.

**Table 1:** Patients' demographics

Author	Year	Time frame	Number of specimens	Site	Patients' age (mean ± SD)	Amount of tissue
Palmer 37	2013	Not mentioned	34 (17 male)	Tibia: 19; femur: 12; humerus: 3	49 years (range, 18–71 years)	1 mm^3^ biopsies
Koga 18	2013	Not mentioned	7	Not mentioned	Not mentioned	“Small amount”
Zimmermann 22	2012	March 2006 to May 2007	8	Humerus: 3; femur: 3; tibia: 2	48.75 ± 9.63 years	10 mm × 10 mm × 10 mm
Gille 38	2012	November 2009 to March 2010	23 (15 male)	Tibial shaft	47.4 years (range, 20–82 years)	At least 3, each measuring 1 cm^3^
Fajardo* 14	2010	August 2007 to March 2008	20 (14 male)	Femur: shaft – 2, subtrochanteric – 2, distal – 2; tibia: shaft – 2, proximal – 1, distal – 1; fibula: shaft – 3; clavicle: midshaft – 4; humerus: proximal – 1; ulna: shaft –2	46 years (range, 32–80 years)	Approximately 5 mg
Kwong† 35	2009	Not mentioned	7 (compared to 8 patients with uneventful healing)	Extra-articular fractures	Range, 18–87 years	Not mentioned
Iwakura 30	2009	Not mentioned	7 (6 male)	Femoral diaphysis: 3; tibial diaphysis: 2; humeral diaphysis: 1; ulnar diaphysis: 1	53.0 years (range, 37–74 years)	“Small amount”
Fajardo* 39	2009	August 2007 to March 2008	15 (11 male)	Femur: shaft – 2, subtrochanteric – 2; tibia: shaft – 2, tibial plateau – 1, distal – 1; fibula: shaft – 2; clavicle: midshaft – 3; humerus: proximal – 1; ulna: shaft –1	46 years (range, 32–80 years); SD 14 years	Not mentioned
Bajada 40	2009	Not mentioned	8 (3 male)	Femur: 5; tibia: 3	55.6 years (range, 26–73 years)	Ranging in wet weight from 120 to 250 mg; mean 162.1 mg
Qu 41	2008	Not mentioned	15 (14 male)	Scaphoid bone	29 years (range, 17–56 years)	>1 mm and up to 3 mm of abnormal bone on either side of the non-union
Hofmann 42	2008	Not mentioned	10 (4 male) compared to 10 (5 male) patients with uneventful healing	Femur: 5; humerus: 3; ulna: 1; pelvis: 1	Non-unions: 59.3 ± 20.3 (range, 25–87 years); Controls: 55.3 ± 15.1 (range, 28–75 years)	Not mentioned
Bajada 43	2007	2004	1 (male)	Tibia	34 years	Not mentioned
Kilian 52	2004	Not mentioned	7 (4 male)	Tibia: 4; humerus: 1; radius: 1; ulna: 1	37 years (range, 32–42 years)	Not mentioned
Reed 44	2002	1993–1999	11 (9 male)	Extra-articular fractures. Tibia: 7; femur: 2; fibula: 1; radius: 1	44 years (range, 14–74 years)	All biopsies >5 mm × 5 mm × 5 mm
Reed 44	2002	1993–1999	11 (8 male)	Extra-articular fractures. femur: 8; tibia: 3	51 years (range, 35–81 years)	All biopsies >5 mm × 5 mm × 5 mm
Kloen 45	2002	Not mentioned	17 non-unions; 4 delayed unions	Humerus: 12; femur: 5; tibia: 2; clavicle: 2	61 years (range, 30–85 years)	Not mentioned
Guerkov 46	2001	Not mentioned	7 (atrophic group: 1 male; hypertrophic group: 2 male)	Femur: 3; clavicle: 2; tibia: 1; iliac wing: 1	61 years (range, 30–85 years)	>0.5 cm^3^
Lawton† 19	1999	Not mentioned	12 patients compared to 15 patients with uneventful healing	Not mentioned	Normal healing: range, 18–87 years	Not mentioned
Lawton† 47	1997	Not mentioned	12 patients compared to 15 patients with uneventful healing	Extra-articular long bone fractures	Normal healing: range, 18–87 years	Not mentioned
Santavirta 48	1992	Not mentioned	10 (7 male)	Tibia: 8; humerus: 2	48 years (range, 27–64 years)	Three parallel representative samples, each about 4 × 4 mm
Boyan 49	1992	Not mentioned	1 (male)	Tibia	19 years	Fibrocartilage lying within the fracture gap and periosteal tissue stripped from the edges of the non-union
Quacci 50	1991	Not mentioned	2 (male)	Tibia	18 and 23 years	5 mm biopsy cannula
Milgram 51	1991	Not mentioned	Extra-articular: 41; intra-articular: 54	Extra-articular: tibia: 13; femur: 10; other: 18. Intra-articular: femur: 44; patella: 4; other: 6	Not mentioned	Sample tissue included the whole fracture site (intact piece)
Heppenstall 32	1987	1970–1983	76 (39 males)	Humerus: 29; femur: 23; tibia: 18; clavicle: 3; metatarsal: 1; ulna: 1; radius: 1	39 ± 3 years	Not mentioned
Urist 53	1954	1948–1953	85 (19 biopsies between 2 and 7.5 years)	Tibia	Not mentioned	Not mentioned

* Both studies used the same samples for their analysis.

† All three studies used the same samples for their analysis.

### Studies characteristics

The studies characteristics are outlined in Table2 14,18,19,22,30,32,35,37–53. The definition of non-union varied between studies, but it was generally based on the radiographic appearance and clinical examination. Most of the samples were obtained during revision operations for the treatment of the non-unions.

**Table 2:** Studies' characteristics

Author	Duration of non-union	Classification	Definition of non-union	Isolation of tissue	Cells/material isolation
Palmer 37	10 months	Aseptic/Septic	Radiographic evidence of non-progression of healing for at least 3 months, or lack of healing by 9 months since the initial injury	Intra-operative specimens were collected from removed implants, surrounding tissue membrane and local soft tissue	Culture analysis; Ibis's second-generation molecular diagnostics; bacterial 16S rRNA-based fluorescence *in situ* hybridization (FISH)
Koga 18	11.0 months (range, 9–13 months)	Viable: 2 patients; Non-viable: 5 patients	>9 months had elapsed since the injury, and the fracture had shown no visible progressive signs of healing for 3 months	The non-union site was exposed by careful incision, and care was taken not to contaminate the bone and periosteum	Histological analysis; flow cytometry; cell proliferation; alkaline phosphatase activity assay; ALP mRNA; mRNA analysis; osterix expression; osteocalcin expression; mineralization assay
Zimmermann 22	>9 months	Radiological appearance	>9 months from injury	Pseudarthrotic tissue was collected out of the fracture gap during regular surgical treatment	mRNA isolation; cDNA arrays
Gille 38	10.2 months (range, 6–34 months)	Aseptic	Absence of osseous healing >6 months from injury	Intra-operative biopsy samples	Cultures; PCR
Fajardo 14	16 months (range, 0.5–6 years)	Hypertrophic	Absence of osseous healing >6 months from injury	Multiple tissue samples from: (*i*) the non-union site and (*ii*) mineralized fracture callus from the surrounding region	RNA extraction; synthesis of cDNA; real-time quantitative PCR; western blot assay (only eight samples); immunohistochemistry (only eight samples)
Kwong 35	Range, 1–48 months	Aseptic; only fractures with areas of cartilage were chosen	Absence of osseous healing >9 months after treatment	Fracture biopsies taken at surgery for treatment of malalignment or failure of fixation, as well as acute fractures that were operated upon in a delayed fashion	Immunohistochemical Analysis
Iwakura 30	11 months (range, 9–14 months)	Hypertrophic	>9 months from injury, no visible progressive signs of healing for 3 months	Samples were obtained during revision surgery	Histological analysis; immunophenotyping of non-union cells by flow cytometry; osteogenic induction; chondrogenic induction; adipogenic induction; total RNA extraction and RT-PCR
Fajardo 39	16 months (range, 0.5–6 years)	Hypertrophic	Absence of osseous healing >6 months from injury	Multiple tissue samples from: (*i*) the non-union site and (*ii*) mineralized fracture callus from the surrounding region	RNA extraction; synthesis of cDNA; real time quantitative PCR; western blot assay (only seven samples); immunohistochemistry (only seven samples): by standard technique
Bajada 40	3 years (range, 2–5 years)	Atrophic	Not mentioned	Tissue was excised from the site of non-union between the diaphyseal cortices and below the pseudocapsule	Histological analysis; CD immunoprofiling
Qu 41	36 months (range, 5–156 months)	Not mentioned	Not mentioned	Bone from either side of the non-union and the fibrocartilagenous central regions were harvested during reconstructive or salvage surgery	Immunocytochemical determination of osteocalcin; ALP enzyme assay
Hofmann 42	Non-unions: 2.6 re-operations (range, 2–4 revisions); Controls: 0 re-operations	Hypertrophic	Not mentioned	Endosteal cancellous bone fragments were taken at sites proximal to non-unions during surgery. Control cultures were obtained from healthy individuals from endosteal sites during implant removal after uneventful fracture consolidation	Osteoblast cell viability; formation of alkaline phosphatase-positive (CFU-ALP) and mineralization-positive (CFU-M) colony forming units; global differences in gene expression
Bajada 43	9 years	Hypertrophic	Not mentioned	During operation for grafting	Histology
Kilian 52	Not mentioned	Atrophic	Not mentioned	Patients surgically treated for resection of atrophic non-union and re-osteosynthesis	Immunohistochemistry; qualitative RT–PCR; LightCycler-based relative mRNA quantification
Reed 44	27 months (range, 11–62 months)	Hypertrophic	A fracture that had not healed within the expected time period, with no progression towards healing on successive radiographs	During surgery, biopsies taken of the material in the non-union gap (interfragmentary tissue) and the cortex immediately adjacent to the gap	Histology; immunohistochemistry; assessment of vascularization; assessment of vessel density
Reed 44	34 months (range, 12–60 months)	Atrophic	A fracture that had not healed within the expected time period, with no progression towards healing on successive radiographs	During surgery, biopsies taken of the material in the non-union gap (interfragmentary tissue) and the cortex immediately adjacent to the gap	Histology; immunohistochemistry; assessment of vascularization; assessment of vessel density
Kloen 45	22 months (range, 3.5–120 months)	Not mentioned	Absence of osseous healing >6 months from treatment	At the time of surgery	Histology; immunohistochemistry
Guerkov 46	20 months (range, 6–36 months)	Atrophic: 4; Hypertrophic: 3	Not mentioned	At the time of revision surgery (central portion of the tissue)	Histology; cell proliferation; [3H]-thymidine incorporation; alkaline phosphatase specific activity; osteocalcin production; collagen production; local factor production
Lawton 19	Range, 4–48 months	Not mentioned (presence of callus)	Not mentioned	Specimens of fracture callus from normally healing fractures (1–4 weeks after fracture) or non-unions (4–48 months after fracture)	*In situ* hybridization
Lawton 47	Range, 4–48 months	Not mentioned (presence of callus)	Not mentioned	Specimens of fracture callus from normally healing fractures (1–4 weeks after fracture) or non-unions (4–48 months after fracture)	*In situ* hybridization
Santavirta 48	Range, 4–25 months	8 cases delayed union; 2 cases established non-unions	Not mentioned	Tissue from the area between the diaphyseal cortices below the pseudocapsule	Immunopathology (inflammatory-cell analysis, analysis of matrix metalloproteinases); neuroimmunology
Boyan 49	12 months	Not mentioned	Not mentioned	During surgical treatment	Histomicrograph; photomicrograph; alkaline phospatase activity; Elisa; densitometric analysis of the cytoplasmic dot blots
Quacci 50	8 months	Hypertrophic	Not mentioned	Through a 5 mm biopsy cannula	Light and electron microscopy
Milgram 51	Not mentioned	Not mentioned	Not mentioned	Surgical resections, amputations and a small number of autopsy obtained specimens	Histological analysis
Heppenstall 32	Humerus: 4.3 years, Tibia: 2.7 years	Synovial pseudarthrosis	Synovial pseudarthrosis	Biopsies	Light and electron microscopy
Urist 53	>18 months	Not mentioned	X-rays >18 months showing: a bone defect; false motion; sclerosis of the bone ends; rounding, mushrooming, or moulding of the fracture surfaces; sealing of the medullary canal with compact bone to form functioning false bone surfaces and an apparent arrest of the process of osteogenesis in the fracture gap	During surgical interventions/autopsy	Histological analysis

### Macroscopic structure of non-union tissue

Urist *et al*. was the first to hypothesize the mechanism of non-union based on its macroscopic and microscopic characteristics 53. He reported that white soft tissue was interposed between the bone segments, a finding later supported by other authors 51, and explained this as fibrinoid degeneration of the connective tissue in the interior of the callus 53. With regards to synovial pseudarthrosis, a yellow frond-like material was found interposed between the bone fragments, with clear serous fluid filling this space in aseptic cases, whereas in septic cases murky fluid was present 32.

### Microscopic structure of non-union tissue

#### Histology

The histological findings of non-union tissue are summarized in Table3 18,19,30,32,35,40,43–48,50,51,53. Where relevant information was available, a direct comparison of histological findings between atrophic and hypertrophic non-unions was attempted (Table4) 30,40,43,44,46,50.

**Table 3:** Histology findings

Author	Classification	Histology
Koga 18	Viable: two patients; non-viable: five patients	Fibroblast-like morphologic characteristics
Kwong 35	Aseptic non-unions, only fractures with areas of cartilage were chosen	Healing fractures: all consisted of areas of cartilage and significant woven bone formation. Non-healing fractures: in most, cartilaginous areas were accompanied by the presence of small amount of woven bone, but significant fibrous tissue. No notable differences in cellular morphology in the cartilaginous areas of the fractures between the two groups
Iwakura 30	Hypertrophic	Mainly fibrous tissue and no ossicles. Non-union tissue contained various amounts of fibroblast-like cells. After a 21-day incubation under chondrogenic conditions, cell pellets had a spherical and glistening transparent appearance
Bajada 40	Atrophic	Samples largely consisted of fibrocartilaginous tissue that contained occasional bony islands. In some areas, the excised non-union tissue was well populated by fibroblastic cells, but other areas were largely acellular and consisted mostly of a collagenous extracellular matrix. Areas of vascularization were seen consistently and the presence of osteoclasts within absorption pits was also occasionally notable. After enzymatic treatment to extract cells and their plating out into monolayer culture, the majority of the adherent cells present were stromal in appearance, *i.e*. bipolar and fibroblastic. Occasional multinucleated osteoclasts were also seen in the early cultures, as were cells with a stellate (possessed multiple cytoplasmic processes) or dendritic appearance
Bajada 43	Hypertrophic	Fibrocartilaginous non-union with little evidence of new bone formation and no signs of infection
Reed 44	Hypertrophic	Specimens contained fibrous tissue, fibrocartilage, hyaline cartilage and bony islands. Areas of new bone formation by both endochondral and intramembranous ossification. Morphologically samples appeared well vascularized
Reed 44	Atrophic	Specimens contained fibrous tissue, fibrocartilage, hyaline cartilage and bony islands. Relatively few areas of new bone formation, predominantly *via* the endochondral route. Necrotic bone was more prevalent in the atrophic non-union group. Morphologically samples appeared well vascularized
Kloen 45	Not mentioned	Delayed unions and non-unions: 11/21 specimens had foci of woven bone (having cuboid-shaped osteoblasts lining the osteoid, suggesting active bone formation) surrounded by large areas of fibrous tissue that was interspersed with areas of numerous blood vessels. Ten of 21 specimens had similar areas of fibrous tissue but lacked woven bone. Within the samples that contained woven bone, two patterns of bone formation were observed: (*i*) bone appeared to be forming directly from fibrous tissues; (*ii*) bone seemed to be forming from cartilage. Other observations included scattered lamellar bone fragments surrounded by osteoclasts and a paucity of lining osteoblasts. Some specimens also showed villous projections resembling synovial pseudarthroses with lining cells resembling synoviocytes
Guerkov 46	Atrophic: 4; hypertrophic: 3	Mainly fibrous tissue with organized collagen bundles. No ossicles were seen in any of the sections examined. All sections from atrophic non-unions were oligocellular and contained few vessels, whereas those from hypertrophic non-unions were more cellular, with little evidence of cartilaginous tissue
Lawton 19	Not mentioned (had callus)	Human fracture callus: heterogeneous appearance with several of the elements of normal fracture healing (haematoma, fibrous tissue, woven and compact lamellar bone, and cartilage) being present in close proximity in any one section. Non-union gap: tissues consisted largely of vascularized fibrous tissue or avascular cartilage
Lawton 47	Not mentioned (presence of callus)	Areas of old bone, new bone formation, non-union gap (either fibrous, cartilaginous or both), and an interface between the gap and bony material
Santavirta 48	8 cases delayed union; 2 cases established non-unions	The morphology of the samples was not dependent on the duration of delayed union/non-union. All samples contained connective tissue of varying density, in which tissue fibroblast-like mononuclear cells seemed to predominate. The cellularity varied inside each sample from poorly cellular, tight connective tissue areas to highly cellular strangs with occasional cartilage or bony islets
Quacci 50	Hypertrophic	Light microscopy: non-union tissue was composed of connective tissue, cartilage (had a hypertrophic aspect and frequently presented degenerative aspects) and fragmented osteoid-like trabeculae
Milgram 51	Not mentioned	Extra-articular locations: presence of non-mineralized fibrous or fibrocartilaginous tissue between the ends of the bone at the old fracture site. Also demonstrated a spectrum of clefts at the site of non-union ranging from tiny microscopic spaces within the soft tissue of the non-union to dominant clefts that completely separated the ends of the fracture (*i.e*. frank pseudarthrosis). Intra-articular locations: demonstrated the same sequence of changes occurring in 24 of the cases. However, 30 of them demonstrated no tissues of a fibrous non-union
Heppenstall 32	Synovial pseudarthrosis	Light microscopy (62 patients): hyaline cartilage, synovial-like lining cells, or synovium and fibrous tissue was present
Urist 53	Not mentioned	When healing does not occur <18 months, the interior of the callus is more likely to show: inflammatory and fibrous connective tissue; failure of fibrous tissue to regress; fibrinoid and hyaline degeneration

**Table 4:** Comparison of histological findings between atrophic – hypertrophic non-unions

Type of tissue	Atrophic	Hypertrophic
Fibrocartilaginous tissue	40,44	43,44
Fibrous tissue	44,46	30,44
Cartilaginous tissue	–	44,46,50
Collagenous extracellular matrix/connective tissue	40,46	40,46,50
Bone tissue	No ossicles 46; occasional bony islands 40,44	No ossicles 30,46; bony islands 43,44,50
Necrotic bone	More prevalent 44	–
Bone production	Predominantly *via* the endochondral route 44	Bone formation by both endochondral and intramembranous ossification 44
Cells	Generally oligocellular 46; some areas acellular 40	More cellular 46
	Fibroblastic: majority of cells 40	Fibroblast-like 30
	Osteoclasts: occasionally 40	
	Bipolar cells: majority of cells 40	
	Cells with a stellate (possessed multiple cytoplasmic processes) or dendritic appearance 40	
Vascularization	Well vascularized 40,44; few vessels 46	Well vascularized 44

#### Immunohistochemistry

The immunohistochemical findings of non-union tissue are summarized in Table5 14,19,35,39,44,45,47,48,52. Interestingly, BMP's were present in the non-union tissue, although their expression was reduced 35,39,45. Moreover, matrix metalloproteinases (MMP's) were also reported to be present in the non-union tissue, not localized in a particular cell type or cellular component 14,48.

**Table 5:** Immunohistochemistry findings

Author	Classification	Immunohistochemistry
Fajardo 14	Hypertrophic	MMP-7 and MMP-12 were found to be stained within the substance of the non-union tissue and not localized within a particular cell type or cellular component. Both enzymes were likewise not visualized in the bone callus specimens
Kwong 35	Aseptic non-unions, only fractures with areas of cartilage were chosen	There was a significant reduction in BMP-2 and BMP-14 expression in cartilaginous areas of non-healing fractures compared to healing fractures, but no statistical differences in the endogenous expression of noggin and chordin (BMP inhibitors)
Fajardo 39	Hypertrophic	BMP-7: absent in the non-union specimens but present in the fracture callus specimens. BMP-2: positive immunostaining was restricted consistently to the fibrous tissue of the non-union tissue
Kilian 52	Atrophic	Immunostaining appeared in close vicinity to immature osteoid trabeculae. EDB+ fibronectin immunostaining was negative for scFvL19 antibody
Reed 44	Hypertrophic	No statistically significant difference in median vessel counts between atrophic, hypertrophic and normal unions
Reed 44	Atrophic	No statistically significant difference in median vessel counts between atrophic, hypertrophic and normal unions
Kloen 45	Not mentioned	The most consistent expression was that of BMP-2, BMP-4, and BMP-7 in the osteoblasts lining the newly formed osteoid. The staining was cytoplasmic and, in certain specimens, was specifically located in the Golgi apparatus, illustrating local production of BMP. No correlation between the location of the delayed union or non-union and staining. In the areas of dense fibrous tissue the presence of staining for all BMP isoforms tested was the same as or less than that in the areas close to bone at all time-points after the fracture. Expression of Type IA, Type IB, and Type II BMP Receptors: positive staining was observed in the osteoblasts lining the ossified tissue, in the areas near the ossification sites, and in the fibrous tissue. As observed for the BMP antibodies, there was a trend towards decreased staining in areas remote from bone formation. There was no clear trend between a decreased percentage of positive staining and an increased duration of the non-union. Expression of pSmad1: in the osteoblasts lining the areas of reactive bone formation as well as in osteoclasts, fibroblast-like cells and chondroblast-type cells
Lawton 19	Not mentioned (had callus)	In normally healing fractures, mature osteoblasts on woven bone were negative for MGP mRNA, but positive for osteonectin, osteopontin and osteocalcin mRNA molecules. In non-unions, osteoblasts displayed a novel phenotype: they were positive for MGP mRNA, in addition to osteonectin, osteopontin and osteocalcin mRNA molecules
Lawton 47	Not mentioned (had callus)	In areas of new bone covered by plump osteoblasts, the matrix was either stained uniformly or in a superficial zone, indicating the presence of collagen type III. Fibrous tissue in the fracture gap was also immunostained positively
Santavirta 48	Eight cases delayed union; two cases established non-unions	Most inflammatory cells were CD4 T lymphocytes and their number was always twice that of the CD8 positive cells. Staining for CD11b positive monocyte/macrophages showed in all samples positive cells scattered in the connective tissue stroma with perivascular enrichments. Mast cells were absent or very rare. Almost all resident cells seem to be involved in tissue remodelling as suggested by their content of fibroblast-type MMP-1 and its proteolytic activator MMP-3 or stromelysin, whereas MMP-8 was rare or absent

#### Neuroimmunohistochemistry

Only one study performed neuroimmunohistochemical analysis revealing paucity or total lack of peripheral innervation in the non-union tissue 48.

#### Analysis of vessel density

Blood vessels were present in cases of hypertrophic non-unions, with a varying density (Table6) 44,48,50. When comparing however atrophic and hypertrophic non-union tissue, an interesting finding was that the number of fields containing no blood vessels, some blood vessels and hot-spots, was very similar 44. This was also confirmed with immunohistochemistry studies, where no significant difference was evident in the median vessel count between atrophic/hypertrophic non-unions and normal unions 44. Finally, histological findings confirmed the presence of vascular tissue in both types of non-unions (Table3) 19,40,44,46.

**Table 6:** Tissue examination

Author	Analysis of vessel density	Electron microscopy (Ultrastructural Examination)
Reed 44	The number of fields containing no blood vessels, some blood vessels and hot-spots was very similar in the atrophic and hypertrophic non-union groups	Not applicable
Santavirta 48	Samples mostly consisted of vascularized connective tissue of varying density	Not applicable
Quacci 50	A lot of blood vessels were present in the tissue, often appearing free of blood and occluded by thrombi at different organization stages	Fibroblasts and chondrocytes found in the non-union tissue seemed normal, with a good secretion apparatus. The cell membranes were able to produce matrix vesicles. Hydroxyapatite crystals could be observed in the cell matrix or inside matrix vesicles
Heppenstall 32	Not applicable	(5 patients) Large amounts of surface fibrin. Some cells had profuse rough endoplasmic reticulum and resembled fibrocytes or Type B synovial lining cells. Some of these cells contained prominent lipid droplets and intermediate filaments. There were also phagocytic cells with vacuoles containing granular and cellular debris, resembling to Type A lining cells or monocyte-macrophages. Surrounding the cells were some necrotic cells, clusters of apatite crystals and occasional clumps of collagen fibres infiltrated with more fibrin-like material. Deeper was more densely packed collagen

#### Electron microscopy

Two studies performed ultrastructural examination of the non-union tissue by the means of electron microscopy (Table6) 32,50. In a study by Quacci *et al*., it was found that the non-union tissue contained normal fibroblasts and chondrocytes 50. In addition, Heppenstall *et al*. who examined synovial pseudarthrosis reported large amounts of surface fibrin and densely packed collagen 32.

### Bacteriology of the non-union

Palmer *et al*. analysed 34 samples obtained from patients with non-unions 37. Although eight samples had a positive conventional culture, only four of 34 cases were negative following analysis of bacterial DNA using a combination of Ibis molecular diagnostics and fluorescence *in situ* hybridization techniques. Similarly, Gille *et al*. examined culture negative samples of 23 patients and reported the presence of bacterial RNA following analysis with PCR in two patients (8.7%) 38.

### Evaluation of tissue sample

#### Cell surface protein expression

Three studies performed flow cytometry to determine the presence of specific proteins on the cell surface (Table7) 18,30,40. The non-union tissue was found to be positive for MSC's related markers CD13 30, CD29 18,30, CD44 18,30, CD90 30, CD105 18,30,40 and CD166 18,30, but negative for haematopoietic markers CD14 18,30, CD34 18, CD45 18,30,40 and CD143 18,30.

**Table 7:** Cell surface protein expression

Author	Cell surface protein expression (flow Cytometry)
Koga 18	Strongly positive for the MSC's related markers CD29, CD44, CD105 and CD166 but negative for the hematopoietic markers CD14, CD34, CD45 and CD133
Iwakura 30	Positive for MSC's related markers CD13, CD29, CD44, CD90, CD105 and CD166, but negative for hematopoietic markers CD14, CD34, CD45 and CD133
Bajada 40	Less than 1% of NUSC and BMSC were immunopositive for CD34 and CD45, while 78% ± 14% (mean ± SD) of NUSC and 92% ± 7% (mean ± SD) of BMSC were immunopositive for CD105

MSC: mesenchymal stem cells; NUSC: non-union stromal cells; BMSC: bone marrow stromal cells.

#### Cell senescence

Bajada *et al*. was the only author to report on the cell senescence of non-union stromal cells 40. According to his findings, from passage I onwards, many of the cells developed an appearance that was less bipolar and more spread along with the development of prominent stress fibres. Further passages lead to prolonged culture doubling times (phenotypic changes are consistent with the onset of cell senescence). When examining the proportion of SA-β gal positive cells, that was significantly greater in the non-union stromal cells when compared to the bone marrow stromal cells, but that did not correlate with the patient's age, number of previous operative procedures or time between original fracture and operative management.

### Cultures characteristics

#### Properties

Cell morphology, viability and proliferation are outlined in Table8 18,30,40–42,46,49.

**Table 8:** Cell culture characteristics

Author	Classification	Intervention	Cell morphology	Cell viability (MTT-Test)	Cell proliferation
Koga 18	Viable: two patients; non-viable: five patients	Group A: BMP-7 alone; Group B: BMP-7+ low-intensity pulsed Ultra Sound	Not applicable	Not applicable	No significant difference in the DNA concentration between the two groups on days 3, 5 and 7
Iwakura 30	Hypertrophic	Not applicable	Not applicable	Not applicable	Proliferation capacity of non-union cells was significantly inferior to that of fracture haematoma cells
Bajada 40	Atrophic	Not applicable	Not applicable	Not applicable	Both non-union and bone marrow stromal cells differentiated along each mesenchymal lineage, forming alkaline phosphatase-positive cells (*i.e*. osteoblastic differentiation), oil red O positive cells (adipocytic differentiation) and depositing an extracellular matrix in pellet culture that stained metachromatically with toluidine blue and was immunopositive for type II collagen (chondrogenic differentiation)
Qu 41	Not mentioned	rhBMP-2	Not applicable	Not applicable	Osteoblastic cell populations isolated from bone harvested from the ilium and the three regions of the scaphoid non-unions had similar proliferative capacity
Hofmann 42	Hypertrophic	Not applicable	Although the morphology of confluent cells did not differ between controls and non-unions, there were significantly more bone nodules in the controls group	At day 4 the mitochondrial succinyldehydrogenase enzyme activity was significantly higher in human osteoblast cultures (compared to human non-union osteoblasts), indicating that the number of metabolically active (viable) cells was higher in this group	At 4 weeks, all cultures in both groups were confluent monolayers, and there was no significant difference in cell numbers between the groups
Guerkov 46	Atrophic: 4; hypertrophic: 3	Pulsed electromagnetic field stimulation	Atrophic non-unions: cells formed a uniform monolayer of elongated cells that had few cellular extensions. Hypertrophic non-unions: also consisted of elongated cells, but the cells were more cuboidal, having cellular extensions in a multilayer. After the cells were treated with pulsed electromagnetic field stimulation for 4 days, cells from the atrophic non-unions were small, elongated, or cuboidal, whereas cells from the hypertrophic non-unions were multi-layered, mostly cuboidal and had cellular extensions connecting with adjacent cells. Cells that were not stimulated remained elongated and fibroblastic	Not applicable	Pulsed electromagnetic field stimulation had no significant effect on the proliferation of hypertrophic and atrophic non-union cultures, at any of the times examined
Boyan 49	Not mentioned	BMP (bovine or dog)	Following BMP treatment cells became elongated and more fibroblast like, with no distinct foci of aggregated cells	Not applicable	Incubation with BMP resulted in an inhibition in cell proliferation in periosteal (significant at 2 mg/ml BMP); 3.7-fold inhibition and fibrocartilage cells (significant at 1 mg/ml BMP; fourfold inhibition)

BMP: bone morphogenic protein.

#### Alkaline phosphatase activity assay – messenger RNA evaluation

Alkaline phosphatase activity and messenger RNA (mRNA) evaluation is outlined in Table9 18,19,30,40–42,46,49,50.

**Table 9:** ALP activity and mRNA examination

Author	Classification	Intervention	ALP activity assay	ALP mRNA	mRNA
Koga 18	Viable: two patients; non-viable: five patients	Group A: BMP-7 alone; Group B: BMP-7+ low-intensity pulsed Ultra Sound	The ALP activity of the non-union tissue-derived cells in Group B was significantly higher by 57% and 32% than that in Group A group on days 7 and 14 respectively	In Group B, the expression level of ALP mRNA was significantly up-regulated by 55%, 24%, 50% and 49% compared with the BMP-7-alone group on days 3, 7, 10, and 14 respectively	The expression level of Runx2 mRNA in Group B was significantly higher by 49% and 134% compared with the BMP-7-alone group on days 10 and 14 respectively
Iwakura 30	Hypertrophic	Not applicable	The level of ALP activity under osteogenic conditions was significantly higher than under control conditions on day 21, and ALP activity of non-union cells was significantly higher than that of fracture haematoma cells under differentiated conditions	The expression of ALP under osteogenic conditions was higher than under undifferentiated conditions in the control group	Not applicable
Bajada 40	Atrophic	Not applicable	The ALP activity of the non-union stromal cells cultures appeared markedly lower than that for bone marrow stromal cells cultures	Not applicable	Not applicable
Qu 41	Not mentioned	rhBMP-2	Baseline ALP activity was similar among cell populations isolated from all regions of the scaphoid non-unions and the ilium after 14 days of culture. rhBMP-2 treatment resulted in a significant increase in ALP activity in all groups (proximal: 1.7-fold; central: 2.1-fold; distal: 1.9-fold; iliac: 1.5-fold)	Not applicable	Not applicable
Hofmann 42	Hypertrophic	Not applicable	The comparison of CFU-ALP as an early marker for osteoblast differentiation at day 7 did not show significant differences compared to controls	Not applicable	Not applicable
Guerkov 46	Atrophic: 4; hypertrophic: 3	Pulsed electromagnetic field stimulation	There was a time-dependent increase in ALP specific activity in all cultures that was significant in the cell layers and in isolated cells at 4 days after confluence. Exposure of the cultures to pulsed electromagnetic field stimulation had no effect on the enzyme activity in either the cell layers or isolated cells. At Day 4, enzyme specific activity in the cell layer had increased in pulsed electromagnetic field treated and control cultures by 99% and 90% respectively. The time-dependent increases in the isolated cells were comparable. In addition, no differences between cultures from atrophic or hypertrophic non-unions were observed	Not applicable	Not applicable
Lawton 19	Not mentioned (presence of callus)	Not applicable	Not applicable	Not applicable	Osteoblasts in non-unions: positive for MGP mRNA signal (in the zone of new bone formation and in the interface zone; old bone zone: almost always negative; gap zone: rarely contained osteoblasts). Small and large chondrocytes in non-unions: negative. Small and large chondrocytes in normal fractures: positive for MGP mRNA. Osteoblasts in normal fractures: never detected
Boyan 49	Not mentioned	BMP (bovine or dog)	There was significant reduction in ALP specific activity in matrix vesicles and plasma membranes from human fibrocartilage and periosteal cells incubated with 2 mg/ml BMP (not at 1 mg/ml BMP). As with connective tissue cells, ALP activity in the plasma membrane did not differ from that of the matrix vesicle membranes, before or after the exposure to BMP. Baseline ALP activity in cultures of human periosteal cells was comparable to fibrocartilage cells delivered from human non-union tissue	Incubation with BMP resulted in dose-dependent increase in transcription of ALP	The relative amounts of each type of mRNA differed (ALP, Collagen Type I and II)
Quacci 50	Hypertrophic	Not applicable	Some matrix vesicles presented ALPase activity inside them, but the main enzymatic activity was present outside and strictly connected to the vesicle membrane	Not applicable	Not applicable

BMP: bone morphogenic protein; ALP: alkaline phosphatase; mRNA: messenger RNA; CFU: colony forming units.

#### Osterix

Koga *et al*. has studied the effect of low-intensity pulsed ultrasound on non-union cells cultured with the presence of BMP-7 and reported no significant difference in the expression of osterix 18.

#### Osteocalcin

Osteocalcin expression is outlined in Table10 18,19,30,40–42,46.

**Table 10:** Osteocalcin expression and mineralization assay

Author	Classification	Intervention	Osteocalcin	Mineralization assay
Koga 18	Viable: two patients; non-viable: five patients	Group A: BMP-7 alone; Group B: BMP-7+ low-intensity pulsed Ultra Sound	No significant differences	The intensity of Alizarin Red S staining in the Group B was significantly higher by 30% than in Group A at day 2
Iwakura 30	Hypertrophic	Not applicable	The expression of osteocalcin under osteogenic conditions was higher than under undifferentiated conditions in the control group	After a 21-day incubation under osteogenic conditions, induced non-union cells formed a mineralized matrix (mineralization significantly higher than that of fracture haematoma cells), contrasting with an absence of mineralized matrix under undifferentiated conditions after the same duration
Bajada 40	Atrophic	Not applicable	Not applicable	Although non-union stromal cells elevated their expression of these markers in response to osteogenic stimuli, there was a marked and significant reduction in their capacity to differentiate along an osteoblastic lineage compared to bone marrow stromal cells
Qu 41	Not mentioned	rhBMP-2	All populations had low numbers of osteocalcin-positive cells (7–9%) when grown in the presence of standard medium. There was no statistical difference in the number of osteoblasts between any of the three regions of the scaphoid and the ilium among cells grown under standard conditions, nor was there any correlation between the number of osteoblasts and the duration of the non-union. Cell populations originating from the central fibrocartilagenous part of the non-union had the greatest variability in osteocalcin staining. Significant increases in osteocalcin expression were observed in all groups in response to treatment with rhBMP-2 (ilium: 2.9-fold increase; proximal and distal: 2.3-fold increase; central: 2.0-fold increase)	Cell populations derived from scaphoid non-unions formed an extracellular matrix that showed very little bone nodule formation when maintained in culture for 28 days. Treatment with rhBMP also resulted in a significant increase in the number of bone nodules for all groups (proximal: 3.5-fold; central: 10.5-fold; distal: 4.9-fold; iliac: 3.4-fold)
Hofmann 42	Hypertrophic	Not applicable	Not applicable	The mineralization of extracellular matrix (CFU-M) was very low in human non-union osteoblast cultures that were cultured under the same culture conditions and was significantly less than that in human osteoblast cultures
Guerkov 46	Atrophic: 4; hypertrophic: 3	Pulsed electromagnetic field stimulation	Osteocalcin was expressed at very low levels by the cultures, indicating the fourth passage cultures contained few, if any, committed osteoblasts. Pulsed electromagnetic field stimulation did not affect production of osteocalcin by non-union cells.	Not applicable
Lawton 19	Not mentioned (presence of callus)	Not applicable	Weakly positive in flattened lining cells on lamellar bone. Positive in multinucleate resorptive cells. Consistently negative in endothelial cells.	Not applicable

#### Osteonectin

Osteonectin expression was investigated by Lawton *et al*. 19. Osteonectin was found to be strongly positive in non-cuboidal and induced osteoblasts of early woven bone, as well as cuboidal osteoblasts of later woven bone. Included osteoblasts and flattened lining cells on lamellar bone were only weakly positive, whereas endothelial cells were consistently negative.

#### Osteopontin

Lawton *et al*. investigated osteopontin expression during the different stages of repair 19. Osteopontin was found to be weakly positive in non-cuboidal osteoblasts on early woven bone, and moderately positive in cuboidal osteoblasts on the surface of woven bone later in repair. Multinucleate resorptive cells were associated with a strong signal, in comparison with most flattened cells on the surface of lamellar bone and endothelial cells that were negative.

#### Bone Sialoprotein

Iwakura *et al*. studied the expression of Bone Sialoprotein under osteogenic conditions and found it to be higher in the non-union cells than under undifferentiated conditions in the human dermal fibroblasts (controls) 30.

#### Mineralization assay

Mineralization assay outcomes are outlined in Table10 18,19,30,40–42,46.

#### Dickkopf-1 expression

The expression of Dickkopf-1 (Dkk-1) was studied by Bajada *et al*. 40. According to his findings, both non-union and bone marrow stromal cells secreted Dkk-1 into conditioned medium at comparable levels under control (*i.e*. non stimulated) conditions. However, Dkk-1 levels detected in stimulated non-union stromal cells conditioned medium were markedly and significantly greater than those found in stimulated bone marrow stromal cells cultures.

#### Gene expression

Several authors have examined the expression of different genes in the non-union tissue. A summary of their results is outlined in Table11 14,22,30,42,52 and Table12 47,49.

**Table 11:** Gene expression

Author	General gene expression	Real-time PCR
Zimmermann 22	Genes expressed more than two times than in normal tissue: CDO1; PDE4DIP; COMP; FMOD; CLU; FN1; ACTA2; TSC22D1	Not applicable
Fajardo 14	MMP-7 and MMP-12 mRNAs were significantly elevated in the non-union tissue when compared with local mineralized callus from the same site	MMP-7 and MMP-12 were the only enzymes (of 53 examined) significantly elevated in non-union tissue when compared with local mineralized callus from the same site
Iwakura 30	Not applicable	It showed the expression of mRNA of Col II, Col X, SOX9 and aggrecan chondrogenic conditions after a 21-day induction. Under adipogenic conditions after a 21-day culture period, it showed the expression of LPL and PPAR-g2 (higher than under undifferentiated conditions in the control group)
Fajardo 39	BMP gene expression in healing bone displayed several up-regulated genes between the two tissues	BMP antagonist genes (DRM, follistatin, noggin): increased in non-union tissue when compared to fracture callus tissue. BMP receptors (R1A, R1B, R2): expressed but did not demonstrate any significant differences. BMP-4: up-regulated in non-union tissue when compared to the fracture callus tissue. RNA levels of the BMP antagonists Drm/Gremlin, follistatin and Noggin: up-regulated in the non-union tissues. BMP-7: increased in the fracture callus tissue
Hofmann 42	Gene terms significantly overrepresented in human non-union osteoblast cultures: skeletal development; response to wounding; organ morphogenesis; vasculature development; proteinaceous extracellular matrix; extracellular space; cytokine activity; glycosaminoglycan binding; growth factor activity; insulin-like growth factor binding. Genes significantly down-regulated in human non-union osteoblast cultures: IGF-2, FGF-1, FGF-receptor 2 (FGF-R2), BMP-4, TGF-β2, PDGF, Wnt-induced proteins (WISP2 and 3), β-catenin and prostaglandin E2 receptor EP4	Confirmed the results of the microarray, especially regarding the down-regulation of some genes involved in osteoblast differentiation and bone metabolism
Kilian 52	Not applicable	In qualitative and quantitative RT–PCR, EDA+ fibronectin mRNA was detectable at low levels. in none of the seven non-union samples, EDB+ fibronectin mRNA transcription was detected by qualitative and quantitative PCR

**Table 12:** Collagen gene expression

Author	Intervention	Type I	Type II	Type III
Lawton 47	Not applicable	Signal for procollagen type I mRNA over fibroblasts and over osteoblasts on woven bone was uniformly strong in most non-unions and normal fractures	Not applicable	Non-unions: in the zone of new bone formation and the interface zone, a population of surface and included osteoblasts was strongly positive for the procollagen type III mRNA signal; osteoblasts in the old zone were usually negative, while the gap zone contained osteoblasts only rarely; fibroblasts were frequently positive in the gap zone and interface. Normal fractures: procollagen type III mRNA was seen in the very early granulation tissue, where most of the positive cells were mesenchymal spindle cells (a cell population that includes osteoblast precursors; osteoblasts were in the vast majority negative; small areas of fibrous tissue in which fibroblasts were either negative or weakly positive
Boyan 49	BMP (bovine or dog)	There was no stimulation of Type I collagen message in the non-union fibrocartilage cells. Non-union periosteal cells were found to be more strongly activated by BMP	The increase in mRNA levels of Type II collagen was not significant compared to controls	Not applicable

#### Western blot assay

Western blot assay was used to detect the presence of specific proteins in the tissue under examination. Fajardo *et al*. investigated the presence of MMP's and reported that MMP-7 and MMP-12 were present in both non-union and mineralized callus tissue; however, the signal intensity of both enzymes was stronger in the non-union tissue 14. In another study, he and his team examined the presence of BMP's 39. His finding included: BMP-2 was present in both non-union and mineralized callus tissue; BMP-4 was detected in non-union samples but decreased in healing bone samples; BMP-7 was detected in the healing bone but was absent in the non-union samples.

#### Comparison between atrophic and hypertrophic non-union tissue

Table4 30,40,43,44,46,50 and Table13 30,40,42,44,46 compare the characteristics of tissue obtained from atrophic and hypertrophic non-unions.

**Table 13:** Comparison between atrophic/hypertrophic non-union tissue

Type of analysis	Atrophic	Hypertrophic
Histology	Table4	
Immunohistochemistry/vessel density	No difference in the median vessel count between atrophic/hypertrophic non-unions 44	
Cell surface antigen profile	CD 105 40	CD13, CD29, CD44, CD90, CD105, and CD166 30
	Cells formed a uniform monolayer of elongated cells that had few cellular extensions 46	Also consisted of elongated cells, but the cells were more cuboidal, having cellular extensions in a multilayer 46
Cell proliferation	No significant effect of pulsed electromagnetic field stimulation 46	
ALP activity	No differences between cultures from atrophic or hypertrophic non-unions 46	
Osteocalcin	Low levels 46	Low levels 46; higher than in human dermal fibroblasts 30
Mineralization assay	Reduced compared to bone marrow stromal cells 40	Higher than haematoma cells 30; lower than human osteoblasts (normal healing) 42

#### Effect of interventions to the non-union tissue

Table14 18,41,46,49 outlines the effects of either pulsed electromagnetic field stimulation or BMP's on the non-union tissue.

**Table 14:** Effect of interventions

Author	Koga 18	Qu 41	Guerkov 46	Boyan 49
Type of intervention	Group A: BMP-7 alone; Group B: BMP-7+ low-intensity pulsed Ultra Sound	rhBMP-2	Pulsed electromagnetic field stimulation	BMP (bovine or dog)
Cell morphology	Not applicable	Not applicable	Changed (Table8)	Changed (Table8)
Cell proliferation	No effect	No effect	No effect	Inhibition in periosteal and fibrocartilage cells
[3H]-Thymidine incorporation	Not applicable	Not applicable	No effect	Not applicable
Collagen synthesis	Not applicable	Not applicable	No effect	Not applicable
Transforming growth factor-β1	Not applicable	Not applicable	Effect in a time-dependent manner	Not applicable
Prostaglandin E2	Not applicable	Not applicable	No effect	Not applicable
Alkaline phosphatase activity assay	The ALP activity higher in Group B	Significant increase in all regions	No effect: cell layers or isolated cells. At Day 4, enzyme specific activity in the cell layer had increased in pulsed electromagnetic field treated and control cultures by 99% and 90% respectively (comparable increase)	Reduction: matrix vesicles and plasma membranes from human fibrocartilage and periosteal cells incubated with 2 mg/ml BMP (not at 1 mg/ml BMP). No effect: connective tissue cells, plasma membrane, matrix vesicle membranes
ALP messenger RNA	Up-regulated by 55%, 24%, 50% and 49% compared with the Group A on days 3, 7, 10 and 14 respectively	Not applicable	Not applicable	Dose-dependent increase
mRNA	The expression level of Runx2 mRNA in Group B was significantly higher by 49% and 134% compared with Group A on days 10 and 14 respectively	Not applicable	Not applicable	The relative amounts of each type of mRNA differed (alkaline phosphatase, Collagen Type I and II)
Osterix	No effect	Not applicable	Not applicable	Not applicable
Osteocalcin	No effect	Significant increases in osteocalcin expression in all groups	No effect	Not applicable
Mineralization Assay	Significantly higher by 30% than in Group A at day 2	Significant increase in the number of bone nodules for all groups (proximal: 3.5-fold; central: 10.5-fold; distal: 4.9-fold; iliac: 3.4-fold)	Not applicable	Not applicable
Type I collagen expression	Not applicable	Not applicable	Not applicable	No effect in non-union fibrocartilage cells but increase in periosteal cells
Type II collagen expression	Not applicable	Not applicable	Not applicable	No effect
Glycosaminoglycan	Not applicable	Not applicable	Not applicable	Increase

#### Genetic predisposition to fracture non-union

Several authors have investigated the theory of genetic predisposition to fracture non-union by analysing samples from peripheral venous blood 33,54 or bone callus 55, and comparing them with uneventful healing fractures. Numerous polymorphisms such as those of two specific SNPs (rs1372857, genotype GG and rs2053423, genotype TT) were identified to be associated with an increased risk of developing non-union 33,55,56.

## Discussion

Non-unions represent a significant public health problem and have been associated with devastating consequences for the patients, their family and the society as a whole 57. The mechanism behind the progression of a fracture to a non-union state is multifactorial and as a consequence the treatment can be very challenging. The treatment of non-unions has evolved over the years from prolonged immobilization 53 to the use of biological stimulation and polytherapy. Such a strategy attempts to address all the elements of a compromized fracture healing response 3,31.

With regard to the macroscopic appearance of non-unions, a common finding is the interposition of soft tissue between the bone fragments 51,53. In aseptic non-unions, this tissue is whiter in colour, occasionally surrounded by clear fluid, compared to infected non-unions where this tissue becomes more yellowish and frequently surrounded by murky fluid 32. The experience of the authors confirms the above findings and in fact the macroscopic appearance of the non-union tissue is used as an additional marker for confirming/suspecting an underlying septic process.

Regarding the culture characteristics of the non-union tissue, there was an inconsistency in the reported findings. This may be because of the different types of non-union tissue examined (*i.e*. atrophic and hypertrophic), as well as because of the different topography of the non-unions from where samples were obtained. Finally, the expression of several genes was reported to be different in non-union tissue and controls 14,22,30,39,42,52, a finding suggesting that such differences may contribute to the pathogenesis of non-unions.

Several similarities were reported in the histological analysis of atrophic and hypertrophic non-unions. The main types of tissues involved include fibrous, cartilaginous and connective tissue in varying degree 30,40,43,44,46,50. In atrophic non-unions, bony islands were not always present 30,40,43,44,46,50, whereas necrotic bone was more prevalent 44. Generally, the cellular density of atrophic non-unions was lower compared to hypertrophic non-unions, while some areas were completely acellular 40,46. This suggests a different cellular background, which may correspond to the higher failure rate following revision surgery of atrophic non-unions 31.

More importantly, Iwakura *et al*. showed that tissue derived from hypertrophic non-unions contains MSC's 30, a finding later confirmed by Koga *et al*. 18. Similarly, Bajada *et al*. reported the presence of biologically active cells in atrophic non-union tissue, largely CD34/CD45-negative, CD105-positive, with the potential to differentiate to osteoblastic, adipogenic and chondrocytic lineages 40.

In contrast to the common preconception that atrophic non-unions are relatively avascular and inert 44,58, several authors have confirmed the vascularity of the atrophic non-union tissue 19,32,40,44,46,48,50. In addition, Reed *et al*. reported no significant difference in the vessel density between atrophic non-unions, hypertrophic non-unions and healing fractures 44. This biological finding may be of importance, as it suggests that treatments targeting to the enrichment and restoration of local angiogenesis could be applied as an effective treatment modality in the clinical setting.

Low-grade infection represents a challenge for the treating surgeon, as laboratory markers (such as C-Reactive Protein, erythrocyte sedimentation rate, white blood count) and conventional cultures of intra-operative samples can be negative 37,38. A possible explanation for this phenomenon could be the presence of biofilms (bacteria adhere on implants and tissues around the fracture site, forming matrix-enclosed communities), which are resistant to “normal” concentrations of systemic antibiotics 37. Palmer *et al*. and Gille *et al*. have reported the benefit of utilizing molecular based techniques to identify these infections 37,38. This can be very important, as distinguishing between septic and aseptic non-union is essential for determining the course of treatment. However, limitations of their use in clinical practice include: the fact that single-primer PCR can only detect one target organism 37; concerns for oversensitivity with regard to clinical relevance 37,59 and associated cost implications.

Cell senescence is known to play an important role in healing and tissue regeneration 60. In essence, the senescence of adult stem cells or more differentiated cells present in the non-union tissue may represent one of the main mechanisms of the loss of the regenerative potential, leading to healing impairment 60. As already mentioned, Bajada *et al*. reported that an increased proportion of non-union stromal cells were senescent when compared to bone marrow stromal cells, which did not correlate with the patient's age 40. However, the pathways leading to this genomic damage and the contribution of several factors (such as repeated cellular replication and the consequent cell stress 40) are yet to be determined.

Bone morphogenic proteins are some of the major signalling molecules, promoting the differentiation of MSC's into chondrocytes or osteoblasts 12,13. Kloen *et al*. reported evidence of ongoing BMP signalling in the non-union tissue, where endogenous BMP's, their receptors and molecules involved in their signal transduction were present in the tissue 45. Moreover, others have suggested that imbalance in the expression of BMP's and their inhibitors Drm (gremlin), follistatin, noggin and chordin, might account for the impaired bone forming ability 35,39. When the non-union tissue was cultured in the presence of exogenous BMP, the MSC's differentiated into functional osteoblasts, with an increased bone nodule formation 41,49. Treatments regulating concentrations of BMP's have already been used in clinical practice with encouraging results (such as BMP-2 and BMP-7 31). Future research is needed to investigate the effects of similar agonist molecules or their inhibitors.

Matrix metalloproteinases are proteases that play an important role in bone remodelling and bone repair. When the MMP's or their inhibitors are disrupted, disorders of fracture healing may occur 14. In a study by Fajardo *et al*., MMP-7 and MMP-12 genes were reported to be significantly up-regulated within the tissue of hypertrophic non-unions 14. When the hypertrophic non-union tissue was examined *in vitro*, it was found that the same proteins directly bounded to and degraded BMP-2, a highly osteoinductive agent 14. This action of the MMP's may be responsible for the impaired fracture healing in the case of hypertrophic non-unions, even though the same finding may not correlate to atrophic fracture non-unions.

Several reports suggest that low-intensity pulsed ultrasound treatment stimulates bone healing, although the mechanism behind this remains obscure 61,62. When applying low-intensity pulsed ultrasound in non-union cells cultures, it was found that there was a significant effect on the osteogenic differentiation rather than proliferation of non-union tissue cells 18. In addition, growth factor synthesis and release was stimulated 46. The use of low-intensity pulsed ultrasound can therefore improve union rates and accelerate the healing process.

Dickkopf-1 is a secreted protein acting as an antagonist of the Wnt signalling pathway, suppressing fracture repair by inhibiting osteogenic differentiation 40,63. Bajada *et al*. has compared the levels of Dkk-1 in atrophic non-union stromal cells and bone marrow stromal cells, reporting an increased secretion by the non-union cells, associated with reduced osteoblastic differentiation 40. When they treated the bone marrow stromal cells with recombinant human Dkk-1 or conditioned medium from the non-union cells, the effect on osteogenic differentiation remained inhibitory 40. This finding suggests that Dkk-1 may play an important role in the development of non-unions, however further research is needed to shed more light on the underlying mechanism of an increased Dkk-1 production by non-union cells.

Another important element of progression to non-union that needs to be discussed is genetic predisposition. Several authors have investigated this theory by analysing samples from peripheral venous blood 33,54, and bone callus 55 and comparing them with uneventful healing fractures. Numerous polymorphisms such as those of two specific SNPs (rs1372857, genotype GG and rs2053423, genotype TT) were identified to be associated with an increased risk of developing non-union 33,55,56.

The herein study has some limitations. First, it excludes studies involving experimental animal models. However, the outcome of such studies should be treated with caution, as they cannot be translated directly to the clinical scenarios. Second, there is an inherent inconsistency in defining non-union, and as such the timing of tissue harvesting would be slightly different, which might be responsible for some of the differences reported among similar studies. Moreover, as the term MSC's is fairly recent, studies performed in earlier years used a different terminology for the same cells, such as osteoprogenitors, skeletal stem cells, *etc*. As a result, their findings could not be compared to those of more recent studies.

Strengths of the study include the systematic approach of analysing the results and the detailed careful analysis of the data obtained. Collectively, this manuscript presents our current understanding of the molecular and cellular pathways that can be involved in the development of non-union. Direct recommendations to be applied in the clinical setting cannot be safely made with the available evidence. We deem essential that a widely accepted definition of the timeframe for non-unions should be set allowing an earlier intervention in such cases. The conceptual frame of the “diamond concept” for a successful fracture healing response should be considered in cases where bone repair is desirable 5. Cellular therapies and inductive molecules with scaffolds have a role to play in future treatment strategies, as would do tissue engineering approaches 64. Although still under intense investigation genetic therapy could be another treatment option in the foreseeable future.

## Conclusion

In conclusion, failure of fracture healing and progression to non-union represents a not uncommon clinical complication carrying devastating consequences. The histopathological appearance of non-union tissue between atrophic and hypertrophic non-union indicates that both types of non-unions are not avascular and contain a potentially active population of MSC's. Pathways believed to be involved in their pathogenesis include an imbalance in the expression of BMP's and their inhibitors, and an up-regulated expression of several substances such as that of the MMP's and Dkk-1which can block the BMP and Wnt pathways respectively. Immerging evidence also support a genetic predisposition in this patient group.
